# Why do cancer patients die? A decade of experience on trends, profile, causes, and timing of deaths in India

**DOI:** 10.5588/pha.26.0040

**Published:** 2026-08-24

**Authors:** A. Doshi, A.A. Alwani, S. Cyriac, A. Philip, A. Joy, J. Jose, M. Ramachandran, K. Ajith, A.D. Harries, A. Seshachalam

**Affiliations:** 1 Department of Medical Oncology, Rajagiri Hospital, Kochi, Kerala, India;; 2 International Union against Tuberculosis and Lung Disease (The Union), Paris, France;; 3 The Union South-East Asia Office, New Delhi, India;; 4 London School of Hygiene and Tropical Medicine, London, UK;; 5 Collaborative Medical Oncologist Group, Trichy, India;; 6 Dr GVN Cancer Institute, Trichy, India.

**Keywords:** SORT IT, operational research, mortality in cancer patients, palliative care, end-of-life care

## Abstract

**SETTING:**

Medical oncology department in a tertiary care hospital, South India.

**OBJECTIVE:**

To evaluate trends, profile, causes, and timing of inpatient deaths among cancer patients between 2016 and 2025.

**DESIGN:**

A cohort study using secondary data from electronic medical records.

**RESULTS:**

There were 474 inpatient deaths. Despite an 11-fold increase in annual admissions (119 to 1,355), inpatient mortality rates remained relatively stable (range 4%–10%). Mean age at death was 63 years (+12), 64% were male, 73% had metastases, and 84% had poor performance status. Most patients (78%) were emergency admissions, 41% utilised palliative care services, and 75% consented to no end-of-life support. Leading immediate causes of death were cancer progression (57%), organ failure (21%), and sepsis (15%), and the leading underlying cause of death was cancer progression (83%). Median time from admission to death was 5 days (interquartile range: 2–10). Admission for treatment-related complications (adjusted relative risk [aRR]: 10.9, 95% confidence interval [CI]: 3.7–32.3) and no consent for end-of-life support (aRR: 2.5, 95% CI: 1.1–6.0) predicted treatment-related deaths, while recent systemic therapy (aRR: 0.7, 95% CI: 0.5–0.97) protected against early death.

**CONCLUSION:**

Cancer progression predominates as the cause of inpatient mortality. Despite increasing admissions over 10 years, inpatient mortality rates remained stable. Nevertheless, preventing treatment-related deaths and integrating palliative care early remain critical quality-of-care priorities.

## Graphical abstract

**Figure:**
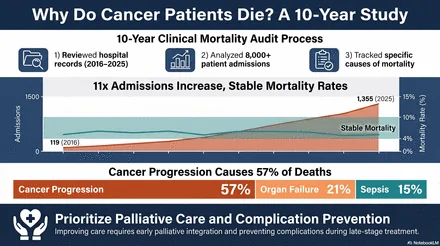

Cancer is a leading cause of mortality worldwide, with a rising global burden. The Global Cancer Incidence, Mortality and Prevalence (GLOBOCAN) report estimated 20 million new cancer cases and 9.7 million cancer-related deaths in 2022, with a rise to 35 million cases by 2050.^1^ Currently, cancer is responsible for one in every six deaths globally.^1^ India contributes substantially to the global cancer burden with an estimated 1.56 million new cases and 0.87 million deaths in 2024.^2^ In recent years, the therapeutic landscape has evolved with the introduction of newer targeted therapies, immunotherapy, and personalised medicine, resulting in improvements in cure and survival rates even in advanced disease.^3^ Despite these advances, overall cancer-related mortality remains high, primarily due to late-stage diagnosis and inequitable access to care.^2^ While cancer mortality is well documented, the immediate causes, timing, and clinical context of inpatient deaths remain poorly characterised, particularly in low- and middle-income countries (LMICs). Understanding whether deaths are driven by disease progression, treatment-related complications, or intercurrent conditions is critical for improving clinical decision making, optimising end-of-life care (EOLC), and identifying preventable factors.

Studies on immediate and underlying causes of death in hospitals are limited and their results are heterogeneous in nature. Most of the data from high-income countries have attributed disease progression as the predominant cause of death (∼77%).^4,5^ However, studies from LMICs have demonstrated variability, with sepsis and multi-organ dysfunction emerging as leading immediate causes in some cohorts, and disease progression predominating in others.^6,7^ Such inconsistencies highlight the need for further robust, context-specific data. Most previous studies on causes of death have been limited by short observation periods and small sample sizes.^5,7,8^ We therefore conducted a comprehensive 10-year study of cancer-related mortality using electronic medical records (EMRs) from a tertiary care oncology centre in South India, with a view to better understand immediate and underlying causes of death.

The study aimed to evaluate the trend, profile, causes, and timing of inpatient death among cancer patients between 2016 and 2025. Specific objectives were to determine: i) the annual trend of admissions and deaths in relation to the COVID-19 pandemic; ii) socio-demographic, clinical, and treatment-related characteristics of those who died; iii) causes, place of death within the hospital, and time to death; and iv) factors associated with treatment-related and early death (within 2 days of admission).

## METHODS

This was a cohort study using secondary data extracted from EMR spanning 10 years (January 2016 to December 2025).

### General setting

The study was conducted at Rajagiri Hospital, Kochi, Kerala, South India. This private hospital is a 567-bed tertiary care referral centre catering to patients across South India. It maintains comprehensive, digitised patient records through an integrated Hospital Information System. The hospital is recognised as a European Society of Medical Oncology–designated centre of integrated oncology and palliative care.

### Specific setting

The Medical Oncology department collaborates with radiation and surgical oncologists and supportive care specialists to provide holistic cancer care. The department offers a 26-bed daycare chemotherapy service and a bone marrow transplant unit, and is supported by 24/7 intensive care unit services. A comprehensive cancer registry has been systematically maintained since 2016. Over 10 years, from 2016 to 2025, the department has recorded over 31,000 daycare admissions and over 8,000 inpatient admissions. The hospital has a dedicated, multidisciplinary pain and palliative care unit providing EOLC, hospice services, and home-based care. Patients are referred by oncologists for palliative care services, after which care is co-managed by oncology and palliative care physicians during subsequent admissions when clinically indicated. For all admissions, the treating physicians’ team updates medical notes daily. When a patient dies, a death summary is recorded by the team, which includes the sequence of events leading to death, including underlying and immediate causes of death. This information is entered in the EMR. A death certificate (copies of which are kept in the medical records department) is also completed.

### Study population

The study population included all adult oncology patients (≥18 years) with a confirmed cancer diagnosis who were admitted under the Medical Oncology Department, Rajagiri Hospital, and who died during their hospitalisation between 1 January 2016 and 31 December 2025.

### Variables, data sources, and data collection

Variables included: demographic, clinical, and disease-related characteristics, including comorbidities and Eastern Cooperative Oncology Group (ECOG) performance scores; final admission details and hospital support needed; treatments given since diagnosis and in the last 30 days; palliative care consultation and EOLC consent (defined as consent to not receive any active life support, including resuscitation); causes of death, stratified by immediate and underlying causes, and early (within 2 days of admission)^9^ and late death; within hospital place of death; and time to death.

### Operational definitions of cause of death

These include^10^: Immediate – the final disease, injury, or complication directly leading to death; Underlying – the disease/injury/event initiating the sequence of events leading directly to death; Cancer-related – death directly attributable to progression or complications of the cancer itself; Treatment-related – death resulting from complications or adverse effects of therapeutic interventions used to treat the cancer.

### Data sources

The data source was the hospital’s EMR system. To determine cause of death, an investigator (AD or JJ) reviewed the treating physician’s admission notes, daily progress notes, and death summary available in the EMR. If the investigator was unable to determine the cause of death from these records because of insufficient information, the death certificate was reviewed. Completed death certificates were available for all inpatient deaths. When multiple immediate causes of death were recorded, only the primary cause was included. If the investigator was uncertain about the cause-of-death classification, the case was reviewed jointly by both investigators. In cases of inter-reviewer disagreement, the case was reviewed by a third investigator (SC).

### Data collection

Data were systematically extracted from the EMR by AD and JJ, and entered directly into EpiCollect5 (V87.2.1, Centre for Genomic Pathogen Surveillance, 2025). To preserve confidentiality and anonymity, unique study identifiers were automatically assigned to each patient within EpiCollect5, and these were de-linked from the clinical data.

### Analysis and statistics

Data were analysed using Jamovi (version 2.6.44). Continuous variables were presented as means (standard deviation [SD]) or medians (interquartile range [IQR]). Categorical variables were reported as frequencies and percentages. Temporal trends in admissions and deaths over 10 years were visualised using line graphs. Univariate analyses were performed using log-binomial regression to determine socio-demographic, clinical, admission, and treatment-related factors associated with treatment-related death and early death. Variables with *P* values <0.1 were included in the multivariable models. For the multivariable analysis of treatment-related deaths, the log-binomial model failed to converge so Modified Poisson regression with robust standard errors was used. Unadjusted and adjusted relative risks (RR) with 95% confidence intervals (CIs) were used as measures of association. Significant levels were set at 95% (*P* < 0.05). Results were reported in line with Strengthening the Reporting of Observational Studies in Epidemiology (STROBE) guidelines.^11^

### Ethical statement

Ethics approval was obtained from Rajagiri Hospital Institutional Ethics Committee (RAJH/2025/148; date: 6 September 2025). Since this study used secondary data, the requirement for written informed consent was waived by the ethics committee.

## RESULTS

There were 8,024 inpatient admissions and 474 inpatients who died between 2016 and 2025.

### Annual trend of admissions and deaths

Over 10 years, admissions increased from 119 in 2016 to 1,175 in 2025, peaking in 2023 (N = 1,355;≈11-fold increase). The greatest increases occurred during and after the COVID-19 period. Despite this, mortality rates remained stable, ranging from 4% to 10% (Figure).

**FIGURE.**
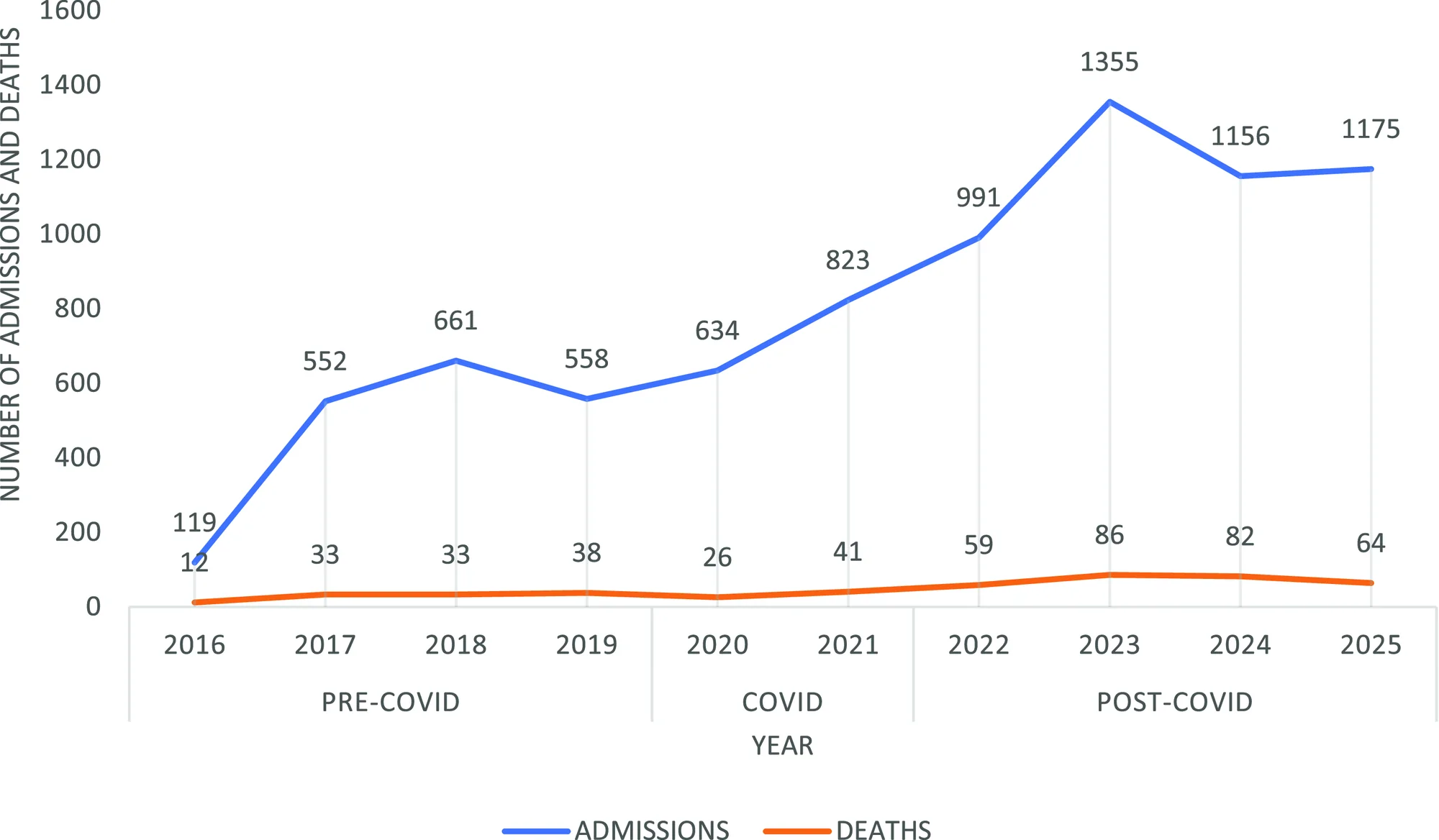
Annual trends of inpatient admissions and deaths among cancer patients admitted to the Medical Oncology Department of a tertiary care hospital in South India (2016–2025).

### Characteristics of inpatients who died

Socio-demographic and clinical characteristics are shown in Table 1. The mean age (SD) was 63.4 years (12.0). Approximately two thirds of patients were male (64%) and from urban areas (71%). Most malignancies were solid tumours, with adenocarcinoma (48%) being the most frequent histological type. Three quarters of patients presented with advanced disease associated with metastases. Comorbidities were common, occurring in 75% of patients. Most patients had poor functional capacity, with 84% having an ECOG performance score of 3 or 4.

**TABLE 1.** Socio-demographic and clinical profile of patients with inpatient mortality admitted to the Medical Oncology Department of a tertiary care hospital in South India (2016–2025).

Characteristics	Variables	Number (%)
Total		474 (100)
Demographic characteristics		
Age (years)	18–<30	6 (1.2)
	30–<60	153 (32.3)
	60–<75	244 (51.5)
	>75	71 (15.0)
Gender	Men	301 (63.5)
	Women	173 (36.5)
Residence **^A^** (N = 471)	Urban	335 (71.1)
	Rural	136 (28.9)
Disease-related characteristics		
Cancer type	Solid tumour	401 (84.6)
	Haematological malignancy	73 (15.4)
Histological type	Squamous cell carcinoma	40 (8.4)
	Adenocarcinoma	226 (47.7)
	Other **^B^**	208 (43.9)
Stage at diagnosis **^A^** (N = 419)	Early (I, II)	33 (7.9)
	Advanced (III, IV)	313 (74.7)
	Not applicable **^C^**	73 (17.4)
Stage at last admission **^A^** (N = 452)	Early (I, II)	4 (0.9)
	Advanced (III, IV)	375 (83.0)
	Not applicable **^C^**	73 (16.1)
Metastatic status **^A^** (N = 448)	Metastases	326 (72.8)
	No metastases	63 (14.1)
	Not applicable **^D^**	59 (13.1)
Clinical status		
Comorbidity	0	118 (24.9)
	1, 2, 3	325 (68.6)
	>4	31 (6.5)
Performance score (ECOG **^E^**) **^A^** (N = 466)	0, 1	7 (1.5)
	2	68 (14.6)
	3, 4	391 (83.9)

A N for these variables is less than 474 because for the remaining participants, the variables were not recorded.

B Other includes small cell carcinoma of the lung, neuroendocrine tumours, glioblastoma, leukaemia, lymphoma, myeloma, etc.

C Stage at diagnosis and last admission are not applicable for malignancies which are not staged as per the TNM staging, for example, haematological malignancies.

D Metastatic status is not applicable for haematological malignancies.

E ECOG = Eastern Cooperative Oncology Group: higher the score, the worse the functional capacity.

Admission characteristics are shown in Table 2. Most admissions were emergencies (78%) and admitted to a hospital ward/room, although nearly 25% were admitted straight to ICU. Cancer-related complaints were the commonest reason for admission (80%), followed by infection (16%) and treatment-related complications (8%). Nearly half the patients had altered consciousness on admission. Oxygen support was required in over 80% of patients, with inotrope and ventilatory support needed in 28% and 22%, respectively.

**TABLE 2.** Admission characteristics and hospital support of patients with inpatient mortality admitted under the Medical Oncology Department of a tertiary care hospital in South India (2016–2025).

Characteristics	Variables	Number (%)
Total		474 (100)
Admission type	Elective	103 (21.7)
	Emergency	371 (78.3)
Place of admission	ICU	115 (24.3)
	Ward or room	347 (73.2)
	Day care	12 (2.5)
Reason for admission **^A^**	End-of-life care	40 (8.2)
	Routine follow-up	11 (2.2)
	Infection	75 (15.8)
	Treatment-related complications **^B^**	40 (8.4)
	Cancer-related complaints **^C^**	377 (79.5)
	Other	28 (5.9)
Consciousness level at the time of admission **^D^** (N = 472)	Alert	258 (54.7)
	Altered	214 (45.3)
Laboratory parameter at admission **^E^**	Severe anaemia **^D^** (N = 463)	80 (17.3)
	Neutropenia **^D^** (N = 463)	52 (11.2)
	Thrombocytopenia **^D^** (N = 463)	128 (27.6)
	Altered LFT/RFT **^D^** (N = 446)	223 (50.0)
	Abnormal electrolyte levels **^D^** (N = 458)	225 (49.1)
Inotrope support during hospitalisation	Yes	134 (28.3)
	No	340 (71.7)
Oxygen requirement during hospitalisation	Yes	394 (83.1)
	No	80 (16.9)
Ventilatory requirement during hospitalisation	Yes	103 (21.7)
	No	371 (78.3)
ICU admission during hospitalisation	Yes	211 (44.5)
	No	263 (55.5)
Dialysis requirement during hospitalisation	Yes	19 (4.0)
	No	455 (96.0)
Number of admissions in the last 6 months **^D^** (N = 471)	0	124 (26.3)
	1–2	113 (24.0)
	3–6	143 (30.4)
	>6	91 (19.3)

ICU = intensive care unit; LFT = liver function test; RFT = renal function test.

A Multiple answers are possible for the same person, so the total N is more than 474 and percentages add up to more than 100.

B Treatment-related complications include any complication of therapy occurring within 30 days of treatment (may also include infection).

C Cancer-related complaints include symptoms, suffering, or functional impairment caused directly by the tumour itself (primary or by metastasis or by paraneoplastic syndrome).

D The variables were not recorded for some of the participants, so the total N is less than 474.

E Severe anaemia: Haemoglobin level less than 8 g/dL; Neutropenia: Absolute neutrophil count less than 1,000 cells/mm^3^; Thrombocytopenia: Platelet count less than 100 × 10^9^/L; Altered LFT: > 2 × upper limit of normal (ULN) aspartate transaminase/alanine transaminase, or >1.5 × ULN bilirubin; Altered RFT: >1.5 × ULN serum creatinine; Abnormal electrolyte levels: Sodium level <127 mEq/L or >145 mEq/L or a potassium level <3 mEq/L or >5.5 mEq/L.

Treatment-related characteristics are shown in Table 3. Most patients had previously received systemic treatment, alone or with surgery/radiotherapy. About 6% of patients had previously received seven or eight lines of chemotherapy/immunotherapy, and 15% had received more than 60 cycles (including oral metronomic therapies). In the previous 30 days, one third had received systemic therapy, with surgery and radiotherapy being uncommon. Three quarters had given consent not to receive any active life support, including resuscitation, during this admission.

**TABLE 3.** Treatment characteristics of patients with inpatient mortality admitted under the Medical Oncology Department of a tertiary care hospital in South India (2016–2025).

Characteristics	Variables	Number (%)
Total		474 (100)
Previous treatment received **^A^** (N = 413)	Surgery and systemic treatment **^B^**	41 (9.9)
	Systemic treatment only **^B^**	182 (44.1)
	Systemic treatment and radiotherapy **^B^**	102 (24.7)
	Other **^C^**	88 (21.3)
Number of lines of systemic therapy **^B^** received previously **^D^** ^**,**^ **^A^** (N = 393)	1–2	251 (63.9)
	3–4	91 (23.2)
	5–6	28 (7.1)
	7–8	23 (5.8)
Number of cycles of systemic treatment **^B^** received previously **^D^** ^**,**^ **^A^** (N = 393)	1–15	259 (65.9)
	16–30	60 (15.3)
	31–60	16 (4.1)
	61–100	58 (14.7)
Any radiation in the last 30 days **^A^** (N = 469)	Yes	28 (6.0)
	No	441 (94.0)
Any surgery in the last 30 days **^A^** (N = 470)	Yes	9 (1.9)
	No	461 (98.1)
Any systemic treatment in the last 30 days **^A^** (N = 467)	Yes	150 (32.1)
	No	317 (67.9)
Received care from the palliative unit	Yes	195 (41.1)
	No	279 (58.9)
End-of-life care consent given	Yes **^E^**	354 (74.7)
	No	120 (25.3)

A The variables were not recorded for some of the participants, so the total N is less than 474.

B Systemic treatment includes chemotherapy, immunotherapy, targeted therapy, and hormonal therapy.

C Other includes only surgery, only radiotherapy, surgery + radiotherapy + systemic treatment, and surgery + radiotherapy.

D A line of therapy refers to a particular treatment regimen used until it is completed, stops working, or is changed. A cycle of therapy refers to one repeated treatment period within a regimen (e.g., Chemotherapy A given for colon cancer is one line, and it is given to the patient in multiple 2-weekly cycles).

E Yes refers to those individuals for whom consent was given (by self or by relatives) to not receive any active life support, including resuscitation.

### Causes, place, and time of death

The predominant immediate cause of death was cancer progression (57%), followed by organ failure (21%) and sepsis (15%) (Table 4). The most common underlying cause of death was cancer-related (83%). About two thirds of patients died in the general ward/room. Median time (IQR) from diagnosis to death was 376 days (116–854), while among patients who received chemotherapy within the preceding 30 days the median interval from last chemotherapy to death was 17 days (9–25). The median time from admission to death was 5 days (2–10).

**TABLE 4.** Causes of death and place of death among patients with inpatient mortality admitted under the Medical Oncology Department of a tertiary care hospital in South India (2016–2025).

Characteristics	Variables	Total, N **^A^**	Early death, **^B^** n (%) **^A^**	Late death, **^B^** n (%) **^A^**
Total		474	144 (30.4)	330 (69.6)
Immediate causes of death				
Cancer-related deaths	Relapse	5 (1.0)	0 (0)	5 (1.5)
	Progression	272 (57.4)	88 (61.1)	184 (55.8)
Other causes of deaths	Bleeding	3 (0.6)	0 (0)	3 (0.9)
	Sepsis	70 (14.8)	23 (15.9)	47 (14.2)
	Lung related	22 (4.6)	5 (3.5)	17 (5.2)
	Cardiac cause	45 (9.5)	9 (6.2)	36 (10.9)
	Renal failure	14 (3.0)	5 (3.5)	9 (2.8)
	Liver failure	20 (4.2)	7 (4.9)	13 (3.9)
	Other **^C^**	23 (4.9)	7 (4.9)	16 (4.8)
Underlying causes of death	Cancer related	393 (82.9)	126 (87.5)	267 (80.9)
	Treatment related	29 (6.1)	7 (4.9)	22 (6.7)
	Other	52 (11.0)	11 (7.6)	41 (12.4)
Place of death	Room/ward	301 (63.5)	90 (62.5)	211 (63.9)
	Intensive care unit	172 (36.3)	53 (36.8)	119 (36.1)
	Day care	1 (0.2)	1 (0.7)	0 (0)

Cause of death was determined from admission, inpatient, and death notes recorded by the treating physician. If the cause of death could not be ascertained from these notes, death certificates were reviewed. Only one immediate and one underlying cause of death is recorded.

A Column percentages.

B Early death refers to death occurring within 2 days of admission. Late death refers to death occurring more than 2 days after admission.

C Other include multi-organ dysfunction syndrome, aspiration pneumonia, seizures, acute stroke, diabetic ketoacidosis, haemophagocytic lymphohistiocytosis, and tumour lysis syndrome.

### Factors associated with treatment-related death and early death

On adjusted analysis, factors independently associated with treatment-related deaths were admission for treatment-related complications (adjusted relative risk [aRR]: 10.9, 95% CI: 3.7–32.3, *P* < 0.001) and no consent for EOLC (aRR: 2.5, 95% CI: 1.05–6.0, *P* = 0.04). One factor that was independently protective of early death was recent systemic treatment within 1 month of death (aRR: 0.7, 95% CI: 0.5–0.97, *P* = 0.03). There were no other significant associations. Adjusted analyses tables are presented in https://figshare.com/s/60397be8583f9f836019.

## DISCUSSION

This study represents one of the largest and longest (10-year) hospital-based EMR analyses providing valuable insights into the clinical trajectory before death, causes, and time to death metrics of cancer patients.

In our study, despite a nearly 11-fold increase in admissions over a decade, mortality rates remained stable. Notably, even during the COVID-19 pandemic, when an upward trend in admissions occurred, mortality rates did not increase. This finding contrasts with other reports from Indian facilities that have described higher mortality among cancer patients during the pandemic.^12,13^ This may reflect better care as well as potential differences in case capture, with patients having severe infections being managed under other specialities.

The most common immediate cause of death in our cohort was cancer progression. This finding aligns with reports of inpatient cancer mortality from high-income countries,^4,5,14^ and some LMICs.^8^ In contrast, in other low-and middle-income settings, infections and sepsis remain dominant causes of death,^6,15^ possibly due to limitations in health care infrastructure, predominance of haematological malignancies, late presentation, delayed diagnosis, and restricted access to health care. Our findings may be explained by the tertiary care setting of our institution, and the relatively strong regional health indicators, including higher literacy rates and better health care access.^16^ Additionally, our cohort predominantly comprised patients with solid tumours who have a lower risk of treatment-induced neutropenia, and therefore sepsis, compared to patients with haematological malignancies.^17,18^

Our cohort mainly comprised patients with advanced disease: four fifths presented in stages III–IV, three fourths consented for EOLC, and almost half received palliative unit care. The predominance of cancer progression as the leading cause of death is consistent with this advanced disease profile. These findings reinforce the importance of integrating palliative care across all levels of oncology care, and support recent calls for its systematic incorporation within cancer centres.^19^ Additionally, the routine use of aggressive intensive care interventions near the end of life for individuals with advanced disease warrants careful reconsideration. Treatment intensity should be aligned with disease trajectory and patient goals, while avoiding potentially non-beneficial escalation of care in advanced cancer. In this context, a lower proportion of ICU deaths among patients with advanced cancer may serve as a meaningful indicator of high-quality EOLC.

Our study offers important temporal information regarding inpatient mortality. Previous Indian hospital–based audits largely reported time to death from admission; one study reported a mean hospital stay of 9 days^8^ and another study reported a median stay of 7 days.^6^ In comparison, our cohort had a median admission-to-death interval of 5 days, last chemotherapy-to-death interval of 17 days, and diagnosis-to-death interval of 12 months, thereby offering a more complete view of the terminal clinical trajectory. The short median duration from admission to death probably reflects the severe clinical status of our patients, with many being admitted as emergencies and nearly half presenting with altered consciousness.

Treatment-related deaths, a potentially preventable cause of death, accounted for a small proportion (6%) of all deaths. To our knowledge, this is among the few studies to identify factors associated with both treatment-related death and early death. Our analysis showed that admission for treatment-related complications and no consent for EOLC support were independently associated with a higher risk of treatment-related death. This suggests that patients presenting with treatment-related complications represent a high-risk subgroup, requiring early recognition and aggressive supportive care to prevent mortality. Additionally, patients without EOLC consent are likely receiving intense chemotherapy regimens, which may partly explain their higher incidence of treatment-related mortality. Patients who received recent systemic therapy were less likely to experience early death, probably reflecting better baseline performance status, closer oncologic follow-up, and timely intervention.

The main strengths of the study were the large sample size, long duration of observation, adherence to STROBE guidelines,^11^ and the use of physician notes and death summaries to ascertain causes of death rather than relying on death certificates, which are prone to inaccuracies in India.^20,21^ There were some limitations. The single-centre tertiary care setting and predominantly urban population may limit the generalisability of the findings. Additionally, the study may be subject to selection bias, as only in-hospital deaths were captured, and haematological malignancies may have been under-represented, as these patients were managed under a separate department. Finally, recording only a single primary cause of death may have overlooked other contributing causes.

Despite these limitations, there are some important take-home messages. First, a small proportion of deaths are treatment-related and potentially preventable with early identification and aggressive management. Second, holistic care is crucial, and embedding proactive palliative care in all levels of the oncology care pathway is essential. Third, this study reinforces the importance of regularly auditing mortality data to provide insights and better identification of preventable causes of mortality.

## CONCLUSION

This large 10-year EMR-based analysis provides one of the most comprehensive characterisations of inpatient cancer mortality in India. Despite an 11-fold rise in admissions, mortality rates remained relatively stable. Cancer progression was the leading immediate cause of death, reflecting advanced disease. Treatment-related deaths, though a minority, were associated with a potentially remediable factor, highlighting opportunities for targeted interventions. These findings provide evidence for strengthening emergency oncology protocols, embedding palliative care earlier in the cancer care pathway, and refining EOLC pathways to ensure that all patients die with dignity, in a setting aligned with their wishes.

## References

[1] Bray F , Global cancer statistics 2022: GLOBOCAN estimates of incidence and mortality worldwide for 36 cancers in 185 countries. CA A Cancer J Clin. 2024;74(3):229-263. 10.3322/caac.21834 38572751

[2] National Cancer Registry Programme Investigator Group . Cancer incidence and mortality across 43 cancer registries in India. JAMA Netw Open. 2025;8(8):e2527805. 10.1001/jamanetworkopen.2025.27805 PMC12368690 40833697

[3] Oluwajembola AM , A review of novel cancer therapeutics and current research trends. Sci World J. 2025;2025:5056618. 10.1155/tswj/5056618 PMC12629702 41267750

[4] O’Brien MER , Mortality within 30 days of chemotherapy: a clinical governance benchmarking issue for oncology patients. Br J Cancer. 2006;95(12):1632-1636. 10.1038/sj.bjc.6603498 PMC2360753 17160081

[5] Bauduer F , Capdupuy C , Renoux M . Characteristics of deaths in a department of oncohaematology within a general hospital. A study of 81 cases. Support Care Cancer. 2000;8(4):302-306. 10.1007/s005209900087 10923770

[6] Prakash G , Characteristics and pattern of mortality in cancer patients at a tertiary care oncology center: report of 259 cases. Asian Pac J Cancer Prev. 2010;11(6):1755-1759. 21338228

[7] Choudhary A , Mortality audit of cancer patients in the department of medical oncology at a tertiary cancer care centre in South India. Cureus. 2024;16(3):e56296. 10.7759/cureus.56296 PMC11018518 38629016

[8] Sundriyal D , Audit of in-hospital mortality from a medical oncology and hemato-oncology center with the emphasis on best supportive care. South Asian J Cancer. 2022;11(2):156-159. 10.1055/s-0041-1739042 PMC9718601 36466983

[9] Farrag A , Osman AM , Ghazaly MH . Pediatric cancer mortality: analyzing early deaths and fatalities in a resource-limited tertiary care context. PLoS One. 2024;19(10):e0312663. 10.1371/journal.pone.0312663 PMC11515956 39466815

[10] Adeyinka A , Bailey K . Death certification. In: StatPearls. Treasure Island, FL, USA. StatPearls Publishing, 2023. 30252271

[11] Von Elm E , The Strengthening the Reporting of Observational Studies in Epidemiology (STROBE) statement: guidelines for reporting observational studies. J Clin Epidemiol. 2008;61(4):344-349. 10.1016/j.jclinepi.2007.11.008 18313558

[12] Seshachalam A , Outcome of COVID-19 in Indian patients with cancer: a multicenter, retrospective study. Cancer Research. Statistics, and Treatment. 2022;5:11-18.

[13] Kuderer NM , Clinical impact of COVID-19 on patients with cancer (CCC19): a cohort study. Lancet. 2020;395(10241):1907-1918. 10.1016/S0140-6736(20)31187-9 PMC7255743 32473681

[14] Oh C-M , Causes of death among cancer patients in the era of cancer survivorship in Korea: attention to the suicide and cardiovascular mortality. Cancer Med. 2020;9(5):1741-1752. 10.1002/cam4.2813 PMC7050076 31960609

[15] Sandill S , Outcomes of cancer patients requiring non-elective medical admission to the intensive care unit of a tertiary care center in India: a 5-year retrospective study. Indian J Crit Care Med. 2026;30(1):13-18. 10.5005/jp-journals-10071-25101 PMC12920298 41726345

[16] International Institute for Population Sciences (IIPS) and ICF . National Family Health Survey-5, India, 2019-21: Kerala. Mumbai, India: IIPS, 2021.

[17] Boccia R , Chemotherapy-induced neutropenia and febrile neutropenia in the US: a beast of burden that needs to be tamed?. Oncologist. 2022;27(8):625-636. 10.1093/oncolo/oyac074 PMC9355811 35552754

[18] Vincent J-L , Sepsis in European intensive care units: results of the SOAP study. Crit Care Med. 2006;34(2):344-353. 10.1097/01.ccm.0000194725.48928.3a 16424713

[19] Salins N , Palliative and end-of-life care initiatives for people dying from cancer in India: a narrative review. ecancer J. 2024;18:1822. 10.3332/ecancer.2024.1822 PMC11959141 40171455

[20] Singh P , A scoping review on the errors in medical certification of the cause of death in India. Indian J Med Res. 2024;160(1):11-21. 10.25259/IJMR_498_23 PMC11463844 39382496

[21] Alipour J , Payandeh A . Common errors in reporting cause-of-death statement on death certificates: a systematic review and meta-analysis. J Forensic Leg Med. 2021;82:102220. 10.1016/j.jflm.2021.102220 34325081

